# Draft Genome Sequence of the Biofilm-Producing *Bacillus subtilis* Strain B-1, Isolated from an Oil Field

**DOI:** 10.1128/genomeA.01163-14

**Published:** 2014-12-11

**Authors:** S. Kesel, F. Moormann, I. Gümperlein, A. Mader, M. Morikawa, O. Lieleg, M. Opitz

**Affiliations:** a Center for NanoScience, Faculty of Physics, Ludwig-Maximilians-Universität München, Munich, Germany; b Faculty of Environmental Earth Science, Hokkaido University, Sapporo, Japan; c Institute for Medical Engineering IMETUM and Department for Mechanical Engineering, Technische Universität München, Garching, Germany

## Abstract

We report here the draft genome sequence of the *Bacillus subtilis* strain B-1, a strain known to form biofilms. The biofilm matrix mainly consists of the biopolymer γ-polyglutamate (γ-PGA). The sequence of the genome of this strain allows the study of specific genes involved in biofilm formation.

## GENOME ANNOUNCEMENT

Biofilm research has become a very important field in microbiology. Due to their high mechanical resilience and resistance to antibiotic treatment, biofilms constitute a significant problem in both industry and health care (1). However, the molecular reason for this outstanding sturdiness of bacterial biofilms is not understood. Several wild-type strains of the Gram-positive model organism *Bacillus subtilis* are known to form biofilms, but they differ in the compositions of the biofilm matrix (2–4). The biofilm-producing *B. subtilis* strain B-1, isolated from an oil field (5), forms thick biofilms, with a biofilm matrix mainly consisting of γ-polyglutamate. Those biofilms have been shown to efficiently absorb multivalent ions from their environment, and this ion absorption in turn leads to an increased stability of those biofilms toward mechanical erosion (6).

The draft genome of *B. subtilis* B-1 was sequenced via Eurofins Genomics (Eurofins MWG GmbH, Ebersburg, Germany). An Illumina standard shotgun library was constructed and sequenced with the Illumina MiSeq platform (Illumina, Inc., San Diego, CA), which produced 120,000 paired-end reads totaling 85 Mbp. The reads were then further processed by *de novo* assembly using the programs Velvet (7) and Newbler (454 sequencing; Roche, Branford, CT), resulting in a scaffold of 3.9 Mbp comprising 68 contigs and a G+C content of 47%. This represents approximately 90% of the whole *B. subtilis* B-1 genome. Subsequent genome analysis was then performed using the programs LAST (8) and BLAST (9).

The newly sequenced genome of *B. subtilis* B-1 was compared to that of the laboratory strain *B. subtilis* 168 (GenBank accession no. AL009126), which resulted in an overall sequence homology of approximately 50%. A direct sequence comparison of several genes important for biofilm formation, namely, *ywsC* (γ-polyglutamate synthesis) (5), *bslA* (surface layer protein) (10), *tasA* (amyloid fiber forming protein) (11), *pel* (structural matrix polysaccharide) (12), and the whole *epsA*-O operon (exopolysaccharide synthesis) (13) was performed. While the gene sequence comparison of *epsH* revealed only a 71% sequence homology, a more significant sequence homology of 82% was found for *ywsC*.

### Nucleotide sequence accession number.

This draft sequence has been deposited at GenBank/DDBJ/EMBL under the accession no. CP009684.
